# Data on morphology, large-scale chromatin configuration and the occurrence of proteins and rRNA in nucleolus-like bodies of fully-grown mouse oocytes in different fixatives

**DOI:** 10.1016/j.dib.2016.03.085

**Published:** 2016-04-01

**Authors:** Kseniya V. Shishova, Yuriy M. Khodarovich, Elena A. Lavrentyeva, Olga V. Zatsepina

**Affiliations:** a Shemyakin–Ovchinnikov Institute of Bioorganic Chemistry, Russian Academy of Sciences, Miklukho-Maklaya Street, 16/10, Moscow 117997, Russian Federation; b Faculty of Bioengineering and Bioinformatics, Lomonosov Moscow State University, GSP-1, Leninskiye Gory, MSU, 1-73, Office 433, Moscow 119991, Russian Federation

**Keywords:** GV oocytes, Nucleolus-like bodies, Fixation procedure, RNA, Proteins

## Abstract

Here we provide data on accessibility of nucleolus-like bodies (NLBs) of fully-grown (GV) mouse oocytes to fluorescence in situ hybridization (FISH) probes and anti-nucleolar antibodies as well as on oocyte general morphology and large scale chromatin configuration, which relate to the research article “High-resolution microscopy of active ribosomal genes and key members of the rRNA processing machinery inside nucleolus-like bodies of fully-grown mouse oocytes” (Shishova et al., 2015 [1]). Experimental factors include: a cross-linking reagent formaldehyde and two denaturing fixatives, such as 70% ethanol and a mixture of absolute methanol and glacial acetic acid (3:1, v/v).

**Specifications Table**

**Table:** 

Subject area	Developmental Biology
More specific subject area	Nucleolus-like bodies (NLBs), fully-grown (germinal vesicle oocytes, GV)
Type of data	Text, figures and tables
How data was acquired	Confocal laser scanning microscopy as described by Shishova et al. (2015) [1]
Data format	Analyzed
Experimental factors	Mouse GV oocytes fixed with a cross-linking fixative paraformaldehyde or two alcohol-containing denaturing fixatives
Experimental features	Phase contrast, chromatin staining with Hoechst 33342, immunofluorescence with anti-nucleolar antibodies, fluorescence in situ hybridization with oligoprobes targeting rRNA, confocal laser scanning microscopy
Data source location	Shemyakin-Ovchinnikov Institute of Bioorganic Chemistry RAS, Moscow 117997, Russian Federation
Data accessibility	Data is provided within this article

## **Value of the data**

• These data demonstrate that the occurrence of nucleolar proteins and RNAs in NLBs should be examined not only after oocyte fixation with paraformaldehyde but also after their fixation with 70% ethanol.
• These data are valuable to researchers interested in investigating the molecular composition of NLBs in mammalian oocytes.

## 1 Data

**Fig. 1:**
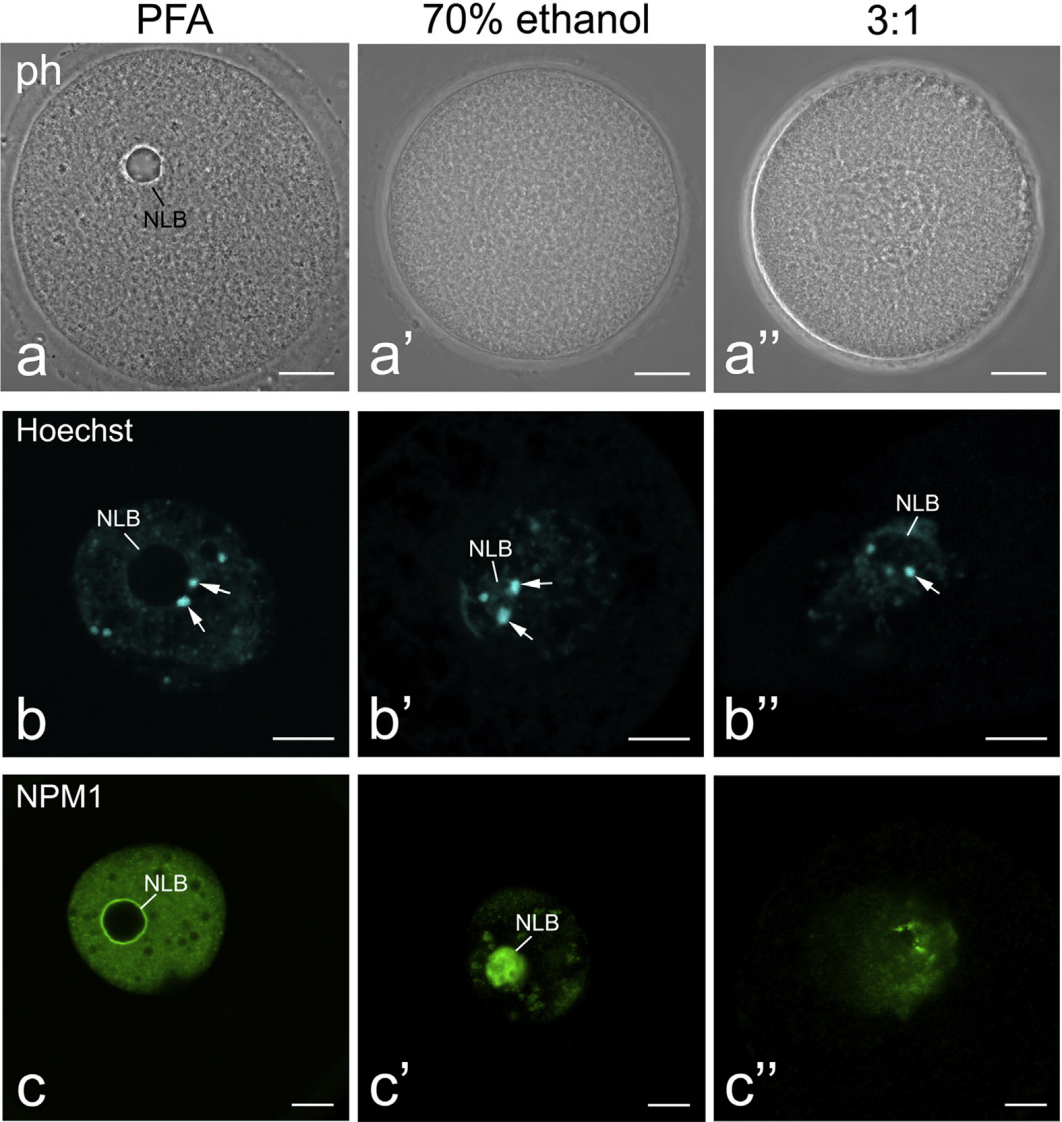
General morphology (a–a″), large-scale chromatin configuration (b–b″) and distribution of a nucleolar protein NPM1 (c–c″) in mouse NSN-oocytes fixed with paraformaldehyde (PFA) (a–c), 70% ethanol (a′–c′) or a mixture of methanol and glacial acidic acid (3:1, a″–c″). In a–a″, the oocytes were examined under phase contrast (ph), in b–b″ – stained with a DNA-binding dye Hoechst 33342, and in c–c″ – processed for immunolabeling with an anti-NPM1 antibody. The denaturing fixatives (a′, a″) cause shrinkage of oocytes, deteriorate general morphology of NLBs and chromatin comparatively with the PFA-fixed oocyte (a). Inside NLBs, NPM1 can be observed only in ethanol-fixed oocytes (c′). NLB – nucleolus-like body. Arrows indicates heterochromatin blocks (chromocenters) typical for the NSN-type oocytes in mice. Scale bars, 10 μm.

**Fig. 2:**
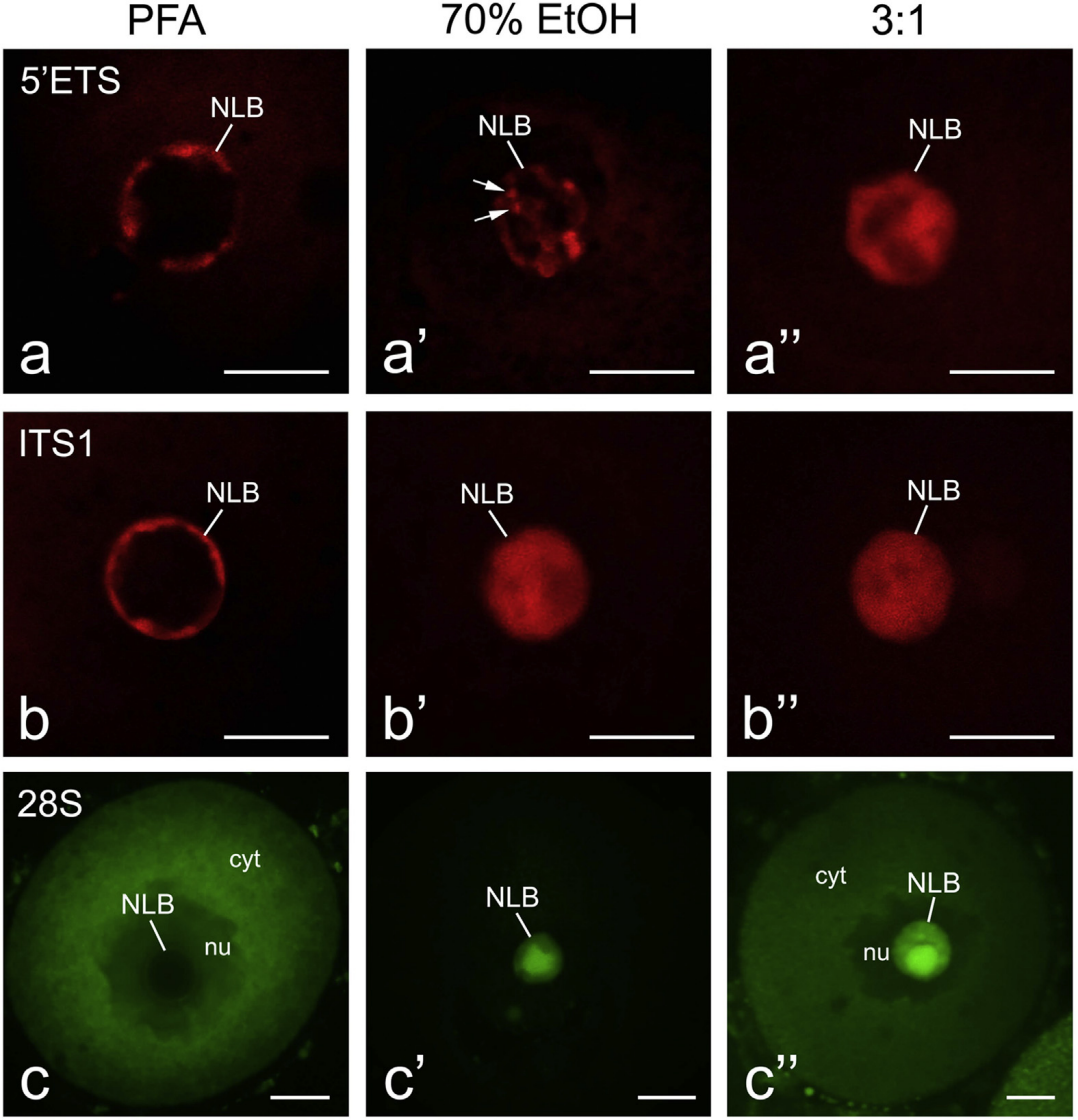
Visualization of the 47S pre-rRNA transcripts (detected with the 5′ETS probe), unprocessed rRNA (detected with the ITS1 probe) and 28S rRNA (detected with the 28S probe) in the NLB mass of mouse GV oocytes of the NSN-type fixed with paraformaldehyde (PFA) (a–c), 70% ethanol (a′–c′) or with a mixture of methanol and glacial acidic acid (3:1, a″–c″) by FISH. The examined rRNAs can be visualized inside NLBs only in oocytes fixed with denaturing fixatives (a′, a″, b′, b″, c′, c″), albeit, unlike 70% ethanol (a′), methanol/acidic acid causes essential redistribution of pre-rRNA transcripts in the NLB mass (a″). NLB – nucleolus-like body, cyt – cytoplasm, nu – nucleus (germinal vesicle). In a′, arrows indicate foci of pre-rRNA synthesis. Scale bars, 10 μm.

Cross-linking (formaldehyde) and denaturing (70% ethanol and methanol/glacial acidic acid, 3:1, v/v) fixatives exert different effects on oocyte and NLB morphology (Fig. 1a, a′, a″), large-scale chromatin configuration (b, b′, b″) and on accessibility of the NLB material to immunofluorescence (c, c′, c″) and fluorescence in situ hybridization (FISH) probes (Fig. 2). Fixation with paraformaldehyde (PFA) best preserves the oocyte phenotype (Fig. 1a) and chromatin configuration (b), but it does not permit to label the nucleolar protein NPM1 (c) and rRNAs (Fig. 2a–c) inside NLBs. In the NLB mass, rRNAs became accessible to different FISH probes [1] only after oocyte fixation with 70% ethanol (Fig. 2a′–c′) or with a mixture of methanol and acidic acid (2a″–c″) despite the mixture can deteriorate resolution labeling as compared with that in the ethanol-fixed oocytes (a′, a″) (Table 1).

## 2 Experimental design, materials and methods

### 2.1 Oocyte collection and fixation

Fully-grown oocytes were collected from C57Bl/6 mice aged 4–8 weeks following the standard hormone administration with PMSG as described in [1]. Oocytes were fixed either with freshly made 3% PFA in PBS (140 mM NaCl, 2.7 mM KCl, 1.5 mM KH_2_PO_4_, and 8.1 mM Na_2_HPO_4_, рН 7.2) or with 70% ethanol in bidistilled water or with a mixture of absolute methanol and glacial acetic acid (3:1, v/v). In all cases, the fixation procedure continued for 20–25 min at room temperature. PFA-fixed oocytes were then treated with 0.5% Triton X-100 in PBS for 10 min, and the other oocytes were exposed to 0.2% Triton X-100 for 5 min to increase accessibility of the used probes and antibodies to target biomolecules.

### 2.2 Phase contrast

Oocytes were fixed with either of three fixatives, placed in PBS and examined as described in Section 2.6.

### 2.3 DNA staining

Large-scale chromatin configuration was examined in oocytes stained with a DNA binding dye Hoechst 33342 (1 µg/ml in PBS) for 10–15 min at room temperature.

### 2.4 Immunofluorescence

Fixed oocytes were washed in PBS (3×10 min), incubated with the mouse monoclonal anti-NPM1 (B23/nucleophosmin) antibody (Sigma-Aldrich, USA, cat. B0556) diluted 1:200 in PBS for 1 h at room temperature, washed in PBS (3×10 min) and placed in Alexa Fluor®488 goat anti-mouse IgGs (H+L) (Molecular probes Inc., cat. A11029) for 45 min at room temperature.

### 2.5 Fluorescence in situ hybridization

Fluorescence *in situ* hybridization (FISH) was performed with antisense oligonucleotide probes recognizing the mouse 47S pre-rRNA. The probe “5′ETS” (5′atc ggg aga aac aag cga gat agg aat gtc tta) hybridizes with the short-lived 5′-external transcribed spacer (5′ETS) segment. The probe “ITS1” (5′ aaa cct ccg cgc cgg aac gcg aca gct agg) hybridizes with the internal transcribed spacer 1. The probe “28S” (5′ gag gga acc agc tac tag atg gtt cga tta) hybridizes with the 28S rRNA sequence (see [1] for the position of the probes along the 47S pre-rRNA). The probes were synthesized by DNA-synthesis Ltd. (Russia), conjugated with Cy3 at the 5′-terminal end and had the stock concentration about 2 μg/μl. Oocytes fixed with either of three fixatives were washed in PBS (3×10 min), then in saline–sodium citrate buffer (2×SSC, 0.3 M NaCl, 0.03 M Na_3_С_6_Н_5_О_7_, рН 7.0; 2×10 min), and placed into the hybridization mix (50% deionized formamide (Sigma-Aldrich), 10% dextran sulfate (Loba Feinchemie GMBH, Austria), 5% 20×SSC (3 M NaCl, 0.3 M Na_3_С_6_Н_5_О_7_, pH 7.0), and 8 ng/µl probes) for 18 h at 42 °C in a wet chamber. Oocytes were sequentially washed with 50% formamide (Panreac, Spain) in 2×SSC (3×10 min) at 42 °C, 2×SSC at 42 °C (10 min), and 2×SSC (10 min) at room temperature.

After DNA-staining, IF and FISH oocytes were mounted in Vectashield® (Vector Laboratories, USA), and examined under a confocal microscope within the next one–two days.

### 2.6 Image acquisition

Eight-bit digital images of oocytes were acquired with a DuoScanMeta LSM510 confocal laser scanning microscope (Carl Zeiss, Germany) equipped with a Plan-Apochromat 63×/1.40 (numerical aperture) **oil Ph3** objective.

## Figures and Tables

**Table 1:** Influence of different fixatives on general morphology, large-scale chromatin configuration and accessibility of the NLB inner material to anti-nucleolar antibodies and FISH probes in mouse GV oocytes.

Fixative/targets	NLB and oocyte morphology (phase contrast)	Chromatin configuration (H33342 staining)	Proteins detection (immunofluorescence)	rRNA detection (FISH)
3% PFA in PBS, 20 min	Excellent	Excellent	No signals	No signals
3% PFA followed by proteinase K^a^	Appropriate	Appropriate	**Excellent**	No signals
70% ethanol, 20 min, on ice	NLBs are undetectable, oocytes shrink	Appropriate	**Excellent/good**	**Excellent**
3:1, 20 min, on ice	NLBs are undetectable, oocytes shrink	Poor	Poor, if any	Appropriate

a According to Shishova et al. [1]. The best conditions for immunofluorescence and FISH analysis of NLBs are made bold.
